# B‐cell memory in malaria: Myths and realities

**DOI:** 10.1111/imr.12822

**Published:** 2019-11-16

**Authors:** Damián Pérez‐Mazliah, Francis M. Ndungu, Racheal Aye, Jean Langhorne

**Affiliations:** ^1^ The Francis Crick Institute London UK; ^2^ York Biomedical Research Institute Hull York Medical School University of York York UK; ^3^ KEMRI/Wellcome Trust Research Programme Kilifi Kenya; ^4^ Department of Immunology and Infectious Disease John Curtin School of Medical Research The Australian National University Canberra Australia

**Keywords:** classical and atypical memory B cells, long‐lived plasma cells, malaria, *Plasmodium*

## Abstract

B‐cell and antibody responses to *Plasmodium* spp., the parasite that causes malaria, are critical for control of parasitemia and associated immunopathology. Antibodies also provide protection to reinfection. Long‐lasting B‐cell memory has been shown to occur in response to *Plasmodium* spp. in experimental model infections, and in human malaria. However, there are reports that antibody responses to several malaria antigens in young children living with malaria are not similarly long‐lived, suggesting a dysfunction in the maintenance of circulating antibodies. Some studies attribute this to the expansion of atypical memory B cells (AMB), which express multiple inhibitory receptors and activation markers, and are hyporesponsive to B‐cell receptor (BCR) restimulation in vitro. AMB are also expanded in other chronic infections such as tuberculosis, hepatitis B and C, and HIV, as well as in autoimmunity and old age, highlighting the importance of understanding their role in immunity. Whether AMB are dysfunctional remains controversial, as there are also studies in other infections showing that AMB can produce isotype‐switched antibodies and in mouse can contribute to protection against infection. In light of these controversies, we review the most recent literature on either side of the debate and challenge some of the currently held views regarding B‐cell responses to *Plasmodium* infections.

## 1 INTRODUCTION

Malaria is a killer disease caused by infection with species of the protozoan parasite, *Plasmodium*. The most deadly of these parasites is *Plasmodium falcipa*rum, for which the estimates of morbidity and mortality in Africa were 219 million and 435 000, respectively, in 2017.^1^ Although several control methods have been employed with substantial success, malaria continues to place a heavy burden on the health systems and economies of the countries affected. Experts agree on the need for the development and subsequent deployment of an effective vaccine, which would be the most cost‐effective means of disease control and could even lead to elimination of malaria. However, despite decades of research in malaria vaccine development, a highly effective vaccine remains elusive. RTS,S/AS01, the most advanced malaria vaccine so far, has 30% efficacy of short‐lived protection.^2^

**Figure 1:**
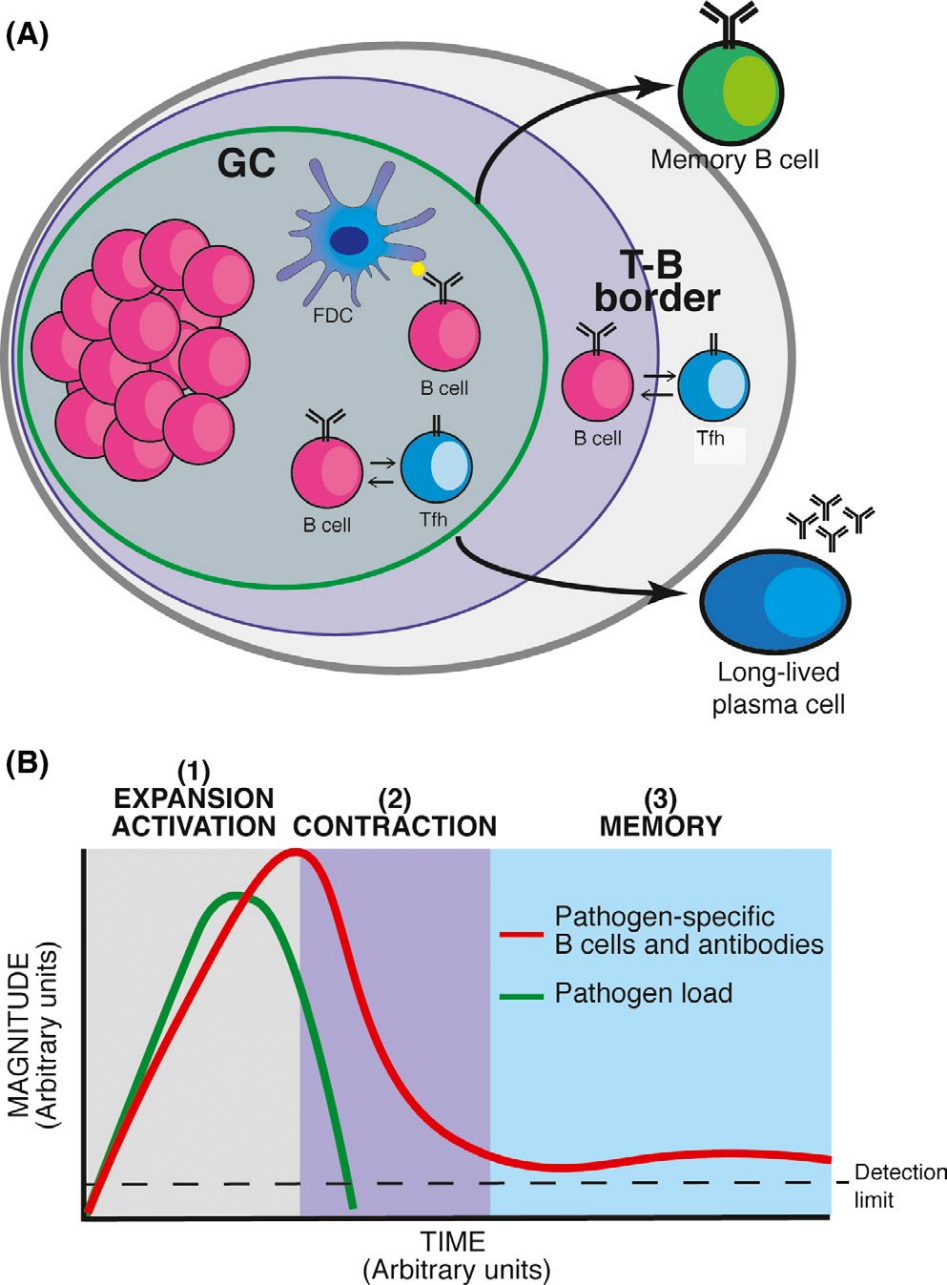
Pathogen‐specific B‐cell responses**. (**A) Schematic representation of the germinal center (GC) B‐cell response, which results in the generation of two arms of B‐cell memory, long‐lived plasma cells and memory B cells. (B) Schematic representation of the kinetics of B‐cell responses to pathogens, showing the expansion/activation phase (1), the contraction phase (2), and the memory phase (3). FDC: follicular dendritic cell; Tfh: follicular helper T cell

A better understanding of the host immune response to malaria, and particularly how to induce and maintain protective levels of circulating antibodies, would be highly valuable for producing effective vaccines which provide long‐lasting protection from malaria. Through extensive studies of humoral responses to immunization with model antigens and in acute viral infections, it is generally accepted that there are two types of long‐lived pathogen‐specific cells of the B‐cell lineage commonly persisting in the memory pool: long‐lived plasma cells, which secrete specific antibodies, in some cases for life; and memory B cells, which confer rapid and enhanced responses to secondary pathogen challenge. Follicular helper T‐cell (Tfh) and germinal center (GC) B‐cell responses are essential to generate isotype‐switched long‐lived plasma cells and memory B cells^3^ (Figure 1A). An understanding of whether and how the immune response is compromised, and of the true longevity of the memory compartments in *Plasmodium* infection is necessary for successful vaccine development.

Protozoan parasites, such as *Plasmodium*, have complex life cycles and, in many cases, different cellular forms within the vertebrate host. The vast majority of protozoan parasites give rise to long‐lasting, and in some cases, lifelong, chronic infections, dramatically shaped by sophisticated immune evasion mechanisms which include a complex and diverse antigenic repertoire. Thus, protozoan parasites represent a substantial challenge to the immune system. Mechanisms that regulate B‐cell responses to *Plasmodium* species have gained increasing attention in recent years. It is now well‐established that B cells and antibodies are critical to control *Plasmodium* infection and to provide immunity to reinfection.^4^, ^5^, ^6^, ^7^, ^8^, ^9^, ^10^ *Plasmodium*‐specific antibodies, in particular of the IgG subclasses, act by inhibiting *Plasmodium* replication and cell invasion, opsonizing extracellular forms as well as infected red blood cells for their destruction by phagocytic cells, and promoting lysis by the complement.^11^, ^12^, ^13^

In contrast, *Plasmodium* infections trigger a series of temporary yet striking events that can potentially alter *Plasmodium*‐specific B‐cell responses. These include pronounced inflammation^14^ polyclonal B‐cell activation and hypergammaglobulinemia,^15^, ^16^, ^17^ alterations in splenic and bone marrow microarchitecture,^18^, ^19^, ^20^ and alteration in hematopoiesis.^21^, ^22^ Moreover, a series of field studies have suggested that B cell responses to *Plasmodium* spp. might be dysfunctional, with poor acquisition of long‐lasting B‐cell responses and accumulation of “exhausted” B cells.^23^, ^24^, ^25^ An intriguing subset of B cells expressing the transcription factor T‐bet, termed AMB, is expanded in subjects exposed to *Plasmodium* infection.^23^ B cells with similar phenotypical characteristics have been observed in response to other infections,^23^, ^26^, ^27^, ^28^ autoimmunity,^29^ and aging.^30^ Whether T‐bet^+^ AMB cells contribute to protection from infection or rather represent a dysfunctional B‐cell subset that leads to parasite persistence and pathology remains a focus of intense debate.

Here, we will review and challenge some currently held views regarding B‐cell responses to malaria, with a focus on the longevity of the circulating antibody response and potential roles of AMB in *Plasmodium* infection in humans and mice.

### 1.1 Are B‐cell responses to malaria short‐lived?

Immunological memory refers to long‐lived immunity sustained in the absence of pathogen re‐exposure. The B‐cell response to a pathogen presents three distinct phases (Figure 1B): (a) Expansion and activation: encounter with the pathogen results in the activation and extensive proliferation of B cells, leading to several fold increase in the frequency of pathogen‐specific B cells as well as the production of pathogen‐specific antibodies by plasmablasts and short‐lived plasma cells; (b) Contraction: as the pathogen load is controlled by the immune response or curtailed by drug treatment, both the frequency of pathogen‐specific B cells and the titer of pathogen‐specific antibodies drop significantly, (c) Memory: following pathogen clearance, a subset of pathogen‐specific long‐lived plasma cells and memory B cells survive the contraction phase; the former continue to produce pathogen‐specific antibodies, sustaining their circulating levels above background; the later recirculate through blood and secondary lymphoid organs readily armed for a second encounter with the same pathogen which initiated the response. Subsequent encounters with the same pathogen have a cumulative effect, resulting in increased precursor frequency of pathogen‐specific memory B cells with each round of exposure. Due to increased frequencies, reduced activation threshold, as well as isotype switching and affinity maturation resulting from GC reactions, the response of memory B cells is typically faster and of greater magnitude compared with that of naive B cells, and results in faster and greater production of antibodies of switched isotypes and increased affinity.^31^

While phases 1 and 2 can typically last for weeks to months, the memory phase can last for years to decades and even for life in the absence of pathogen re‐exposure.^32^, ^33^ Long‐term immunity is a feature of many systemic infections such as mumps, polio, yellow fever, smallpox, measles, and rubella.^32^, ^34^ Studies showed that detectable antibody titers to smallpox could be sustained for over 75 years after a single vaccination,^35^, ^36^ and smallpox‐specific memory B cells could be detected in the blood of vaccinees up to 60 years postvaccination.^37^ Amanna and colleagues performed a longitudinal analysis of antibody titers and memory B‐cell frequencies specific for viral antigens (vaccinia, measles, mumps, rubella, varicella–zoster virus, and Epstein–Barr virus) and nonreplicating antigens (tetanus and diphtheria).^32^ Both antibody responses and memory B‐cell responses were remarkably stable and long‐lived, with antibody responses half‐lives ranging from an estimated 50 years for varicella–zoster virus to more than 200 years for other viruses such as measles and mumps.^32^ Similar longevity of virus‐specific B‐cell memory was observed to natural exposure, as demonstrated by the identification of memory B cells specific for the 1918 pandemic strain of influenza virus circulating in the blood of survivors 90 years after primary exposure.^33^ In comparison, antibody responses to tetanus and diphtheria showed a shorter half‐life (11‐27 years) but still lasting at least a decade, and even over 50 years in some diphtheria cases.^32^, ^35^, ^38^

*Plasmodium* infection*,* with its complex life cycle, antigenic variation/polymorphism, and its ability to establish chronic infection could well influence development and longevity of B‐cell responses, and might not necessarily reflect the kinetics observed after acute viral or bacterial infections or vaccination. In fact, immunity to *Plasmodium* parasites is acquired much less efficiently than in most viral and bacterial infections, where a single infection is enough (sometimes) for life‐long protection.^39^

**Figure 2:**
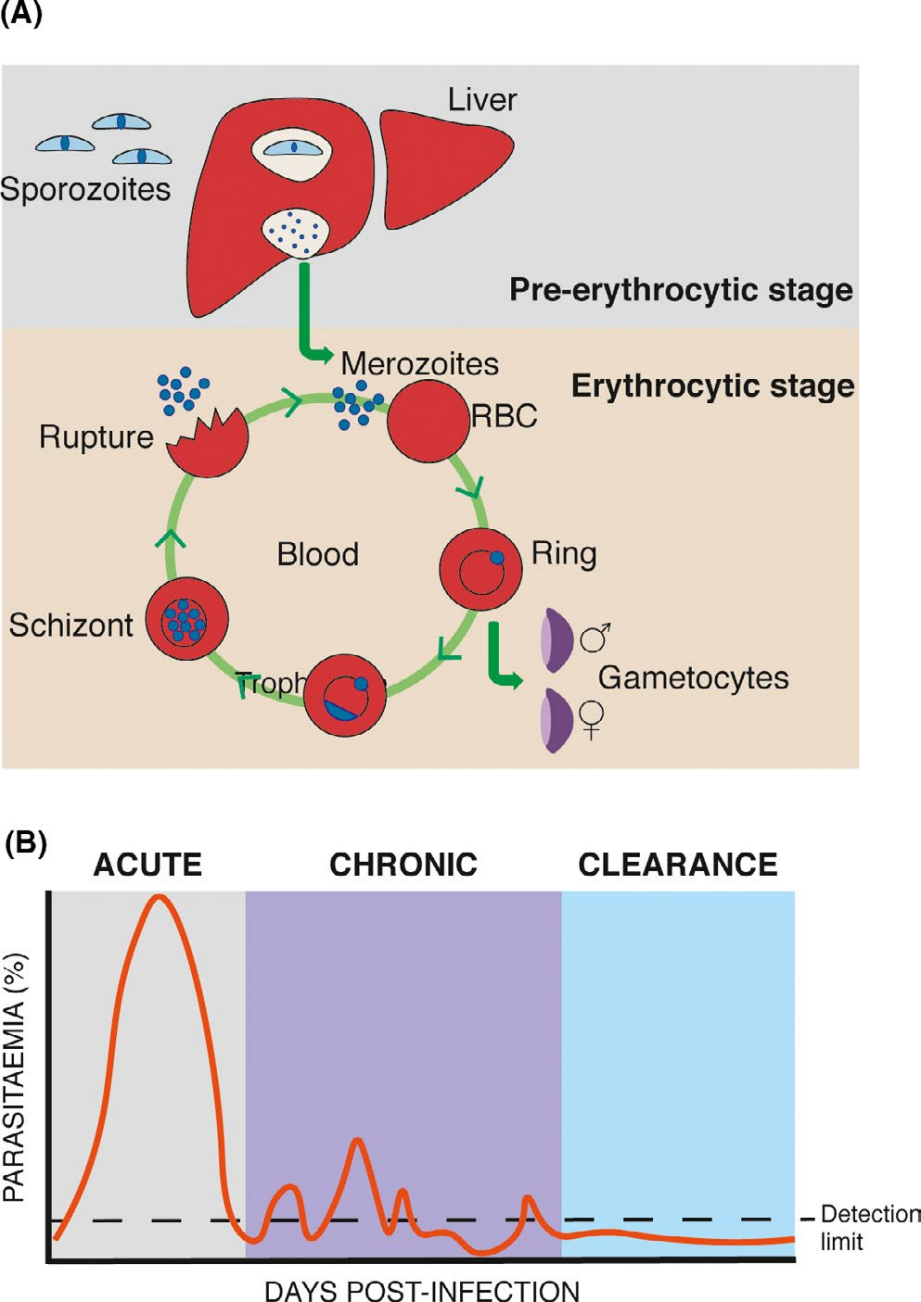
*Plasmodium* infection. (A) Schematic representation of pre‐erythrocytic (liver) and erythrocytic (blood) stages of *Plasmodium* spp. infection in the mammalian host. (B) Schematic representation of the course of erythrocytic *P. chabaudi* infection in C57BL/6 mice

*Plasmodium* parasites present two very distinctive stages in the mammalian host (Figure 2A). An initial pre‐erythrocytic or liver stage, during which *Plasmodium* sporozoites invade hepatocytes. The liver stage can last between 2 and 7 days,^40^ depending on the parasite and host species, and does not lead to clinical disease. The liver stage is followed by a more prolonged erythrocytic stage in which *Plasmodium* merozoites invade red blood cells, giving rise to a continuous cycle of invasion, replication, and cell destruction followed by more invasion, leading to clinical manifestations which in some cases can be severe or even lethal. As mentioned, *Plasmodium*‐specific antibodies confer protection from *Plasmodium* blood‐stage infection in both human and mice.^41^ However, in humans, the presence of *Plasmodium*‐specific antibodies alone seems not sufficient to prevent reinfection.

In areas of stable transmission, *Plasmodium*‐associated life‐threatening disease is very much restricted to young children under 5 years of age and pregnant women.^41^, ^42^ Immunity to these severe clinical manifestations is acquired rapidly, generally after one or two infections, and appears to be maintained in the absence of boosting by reinfection.^43^ In contrast, immunity to infection and non‐severe disease takes several years to develop and requires continuous exposure to the parasite. These observations prompted the hypothesis that B‐cell responses to *Plasmodium* parasites might be dysfunctional or suboptimal, and that generation of long‐lasting humoral memory to the parasite might be defective. In support of this, a series of field studies showed that titers of *Plasmodium*‐specific antibodies drop rapidly after acute infection.^44^, ^45^, ^46^, ^47^ Moreover, persistent infection seems to be required to maintain high titers of *Plasmodium*‐specific antibodies, as reduction of transmission due to insecticide spraying or antimalarial treatment leads to a general reduction of *Plasmodium*‐specific antibodies, albeit not to reach background levels from naive populations. These field studies have an intrinsic limitation in that the source of antibodies cannot be unequivocally determined. Circulating antibodies can be produced either by short‐lived activated plasmablasts/plasma cells or from long‐lived plasma cells as part of a memory response. Thus, a decline in circulating antibodies does not necessarily represent a defect in long‐lived plasma cells. Measurements of the duration of antibody responses are further confounded by likely constant re‐exposure to the parasite in endemic areas. In order to control for these confounding factors, we resorted to study the different compartments of the B‐cell response to *Plasmodium* using mouse models of infection. We have exploited *Plasmodium chabaudi* infections in mice, which present a series of advantages. In particular, similar to human *Plasmodium* infections, *P. chabaudi* erythrocytic infections in mice give rise to an acute phase of infection followed by a distinctive chronic persistent infection with subpatent parasitemia which can last for up to 3 months, thus allowing to study the impact of low‐grade chronic infection on the B‐cell response (Figure 2B). Using this model, we showed that long‐lived plasma cells that secrete anti‐*Plasmodium* antibodies are generated and maintained in the later stages of malaria infections, and that these cells are maintained independently of the low‐grade chronic infection.^48^ In the same study, using in vitro cultures and ELISpot, we showed that a distinctive *P. chabaudi*‐specific memory B‐cell pool survives for a very prolonged period following complete parasite elimination.^48^ More recently, we confirmed this result using ex vivo flow cytometry in combination with natural mosquito transmission.^49^ Thus, a single *Plasmodium* infection in mice leads to the generation of long‐lived plasma cells and memory B cells specific to a dominant *Plasmodium* blood‐stage antigen, which are maintained above naive background level for very prolonged periods of time (presumably for life in mice) independently of the presence of a chronic infection.

These results in mice not necessarily contradict with the results observed in studies of human malaria. As described in Figure 1B, the B‐cell response goes through a dramatic expansion phase shortly after infection, followed by a contraction phase once clearance of the pathogen has begun. Thus, the drop of *Plasmodium*‐specific antibodies after the acute infection observed in some field studies might not necessarily be evidence of poor B‐cell longevity, but rather part of a contraction phase characteristic of short‐lived effector responses following reduction in parasite load or exposure to new antigenic variants (Figure 1B). In agreement with this, we have shown that *Plasmodium*‐specific antibody titers drop rapidly by several fold after *P. chabaudi* infection in mice, but are then sustained above background level for several months.^48^,^50^ Moreover, *Plasmodium*‐specific antibody levels after reinfection display kinetics consistent with secondary antibody responses.^48^, ^50^ Evidence of rapid boosting of antibody titers in response to *Plasmodium* re‐exposure has been largely documented in areas of seasonal malaria transmission, after outbreaks and in travelers.^47^, ^51^, ^52^, ^53^, ^54^, ^55^, ^56^ This is strongly indicative of secondary B cell responses driven by memory B cells. Moreover, although clinical immunity wanes in the absence of exposure, it has been documented that individuals, who are no longer exposed, experience significantly milder forms of the disease and lower levels of parasitemia when compared to fully naive individuals.^57^, ^58^, ^59^ Thus, immunological memory might indeed provide some level of protection. One possible interpretation of these data is that memory B cells contribute to protection from life‐threatening symptoms, while clinical immunity to infection might be largely conferred by short‐lived recently activated antibody‐producing cells sustained by persistent infection and constant re‐exposure. In contrast, the mechanisms that control severe disease might be entirely independent of B‐cell responses and antibodies. Immunity to severe symptoms might be related to a change in the type of immune response, such as a reduction in inflammatory responses or a switch away from a strong Th1 response, as we have shown in mouse models.^60^ In contrast, elimination of infection (mild or asymptomatic malaria) might require antibody responses to all variant forms, something we might expect to take time to develop.

Constant re‐exposure in areas of high transmission is a major confounder in estimating the longevity of B‐cell responses. Thus, evaluating *Plasmodium*‐specific B‐cell responses in historically infected individuals, but who are no longer exposed to malaria might be the most insightful way to measure longevity. In a study of adult Swedish residents who had traveled to malaria endemic areas, *Plasmodium*‐specific memory B cells were detected in 80% of these individuals. These responses were maintained for prolonged periods of time, lasting up to 16 years in the absence of re‐exposure to parasites in some cases. On the other hand, only 30% of travelers showed *Plasmodium*‐specific antibodies above naive levels; some displayed *Plasmodium*‐specific antibodies after at least a decade in the absence of re‐exposure.^61^ In other studies, migrants from endemic areas residing in Spain for long periods (>5 years) without continuous malaria exposure presented seropositivity of 32%‐98% for erythrocytic antigens,^55^ while 88% of migrants residing in France showed *Plasmodium*‐specific antibodies, in some cases for up to 4 years in the absence of re‐exposure.^62^ In studies of malaria outbreaks in Madagascar followed by drastic reductions in transmission, Migot *et al* showed that most exposed individuals maintained *P. falciparum* and *P. vivax*‐specific antibody and memory B cell responses above naive levels for as long as 3‐8 years.^52^, ^53^ In a study of subjects briefly exposed to a *Plasmodium vivax* malaria outbreak in Brazil, outside of the area in which malaria was endemic, *Plasmodium*‐specific antibody titers remained positive in 47% of cases after 7 years.^63^ In a study of adults with previous exposure living in an area of extremely low malaria transmission in Thailand, Wipasa and colleagues documented *P. falciparum* and *P. vivax*‐specific antibody titers and memory B cells which persisted for more than 7 years in the absence of re‐exposure.^64^ Several other studies have documented stable *Plasmodium*‐specific memory B cell^56^, ^65^, ^66^, ^67^, ^68^ and antibody levels^69^, ^70^, ^71^, ^72^ in non‐re‐exposed adults. Moreover, the magnitude and longevity of *Plasmodium*‐specific memory B‐cell responses was shown to be similar to the responses to other classic vaccine antigens such as diphtheria and tetanus toxoid,^64^, ^65^, ^73^, ^74^ suggesting that the immune system can indeed mount memory B‐cell responses to *Plasmodium* antigens to the same extent as to other antigens. Most of these studies have been carried out with adults. However, it seems that previously exposed children who had documented *P. falciparum* infections several years ago, but minimal exposure since, maintain *Plasmodium*‐specific memory B cells at similar levels as compared to those of persistently exposed children living in a separate but nearby endemic area.^75^

In contrast, several field studies, including ours, have shown that *Plasmodium*‐specific antibody responses can be substantially shorter‐lived than their cognate memory B‐cell responses, particularly in children.^46^, ^47^, ^61^, ^75^ Moreover, in some cases, *Plasmodium*‐specific antibody responses were shown to be considerably shorter than tetanus toxoid in the same individuals.^46^, ^47^, ^56^ This would either suggest that while memory B cells can be long‐lived, there might be specific problems in the maintenance of their cognate plasma cells, or that antibodies specific to *Plasmodium* antigens are mostly generated by short‐lived plasma cells not resident in survival niches such as the bone marrow. Similar results have been reported in HIV and HBV, where antigen‐specific memory B cells were found in circulation in the absence of their corresponding antibodies in contemporaneous plasma.^76^ The molecular and cellular basis for this observation is unclear. However, there are two schools of thought on the mechanisms for long‐term maintenance of plasma cells: (a) they could be intrinsically long‐lived or (b) are prone to decaying over time but replenished from the circulating memory B‐cell pool. In the latter case, it has been argued that this could be mediated by restimulation of memory B cells by either antigen retained in the system, or polyclonal stimulants including T cell cytokines and TLR ligands (bystander activation).^46^, ^77^ In addition, environmental factors such as nutritional status and co‐infections can impact longevity of the humoral response. In this sense, pre‐established long‐lived plasma cells seem to be in constant competition for their survival niches with newly recruited plasma cells^77^ and co‐infections with *Plasmodium* parasites and viruses seems to alter humoral responses to both viral^78^ and *Plasmodium* 79 antigens.

Age seems to be another important factor affecting longevity of the humoral response, as adults seem to make better long‐lived antibody responses to *Plasmodium* antigens than children.^44^, ^45^, ^80^ However, it is not clear yet if maturity of the immune system, rounds of re‐exposure, or a combination of both can explain this phenomenon. Finally, low‐transmission regions seem to favor the development of long‐term humoral immunity to malaria.^64^, ^65^, ^72^, ^81^ Thus, *Plasmodium*‐specific memory B cells seem to have a longevity similar to that of memory B cells specific to other antigens. On the other hand, *Plasmodium*‐specific antibody responses may be shorter lived. However, this is not a universal finding,^62^, ^64^, ^65^, ^74^, ^82^, ^83^ and is probably influenced by age of the host, nature of the antigen, intensity of transmission in specific regions, and natural host‐to‐host variations. Identifying the mechanisms that favor the generation of long‐lived plasma cells and consequently long‐lived antibody responses to *Plasmodium* parasites can critically contribute to the development of long‐lived protective malaria vaccines, and therefore should receive major attention.

### 1.2 Are atypical memory B cells “memory,” “effector,” anergic, protective or pathogenic?

An intriguing subset of B cells expressing the transcription factor T‐bet, termed AMB, has been shown to be expanded in blood of subjects exposed to *Plasmodium* infection.^23^ B cells with similar phenotypical characteristics have been observed in response to several other chronic infections including HCV, HIV, *Mycobacterium tuberculosis*, *Toxoplasma gondii,* and *Leishmania infantum.* 28, 84, 85, 86 Moreover, their involvement in immune responses seems to go beyond infections, as B‐cell subsets with very similar phenotypes have been shown to be expanded with age,^30^, ^87^ and suggested to be a driving force in autoimmune disorders.^88^ As these B cells have been identified independently by different research groups working in different fields, they have received a variety of alternative denominations, including “exhausted memory B cells,” “tissue‐like memory B cells,” “age‐associated B cells,” “double negative B cells,” and “T‐bet^+^” or “CD11c^+^T‐bet^+^ B cells.” In this review, we will use the denomination they were originally given in the field of malaria: AMB. Whether T‐bet^+^ AMB contribute to protection from malaria infection, or rather represent a dysfunctional B‐cell subset that leads to parasite persistence and pathology, remains a focus of intense debate.

AMB were first described in the context of malaria by Weiss and colleagues over 10 years ago.^23^ These cells showed a very similar phenotype to a FCRL4^+^ B‐cell subset that was described to be expanded in the blood of HIV‐infected individuals with high viral loads.^26^ More recently, malaria‐associated AMB were shown to preferentially express FCRL5 and FCRL3 but not FCRL4 as previously thought.^24^, ^89^ Similar to HIV, high‐circulating antigen load (parasitemia) seemed to favor the accumulation of this atypical B‐cell subset in malaria, thus suggesting chronic persistent infection may drive the expansion and accumulation of this B‐cell subset in peripheral blood.^23^ In the context of HIV, AMB were termed “exhausted” tissue‐like memory B cells, due to their similarity to a memory B‐cell subset found in human tonsillar tissues.^90^ In addition to FCRL5 and FCRL3, these cells express relatively high levels of other potentially inhibitory receptors including, CD22, CD85j, CD85k, LAIR‐1, CD72, and PD‐1, and show a trafficking receptor expression pattern consistent with a profile of migration to inflamed tissues, which includes CD11b, CD11c, and CXCR3. AMB are antigen‐experienced, class‐switched B cells, which lack the expression of GC markers. Further studies demonstrated the expression of the transcription factor T‐bet and the cytokine IFNγ by these cells, also characteristic of Th1 CD4^+^ T cells.^84^, ^91^, ^92^ Although they received the “memory” denomination, AMB do not express CD21 or the hallmark human memory B‐cell marker CD27, and have a substantially shorter life span than classical CD27^+^ memory B cells.^26^

Comparison of B‐cell profiles from children living in a rural community of *P. falciparum* transmission with those of age‐matched children living under similar conditions in a nearby community where *P. falciparum* transmission ceased over 5 years prior to the study shows that increases in AMB are driven by *P. falciparum* exposure, and not influenced by other factors commonly associated with malaria, such as coinfections and malnutrition.^25^ Moreover, temporary expansion of AMB in blood has been documented in response to human controlled malaria infections.^93^, ^94^ The appearance of AMB is strongly associated with high parasitemias or high exposure to the parasite, as individuals living in areas of high malaria transmission present higher frequencies of AMB than those in areas of moderate transmission.^89^ Similarly, repetitive *Plasmodium* episodes result in higher frequencies of AMB,^92^ and AMB frequencies are greater in children with persistent asymptomatic *P. falciparum* parasitemia compared with parasite‐free children.^23^ Conversely, AMB frequencies decline as parasitemias are reduced or eliminated and when there is no further exposure^61^, ^74^, ^95^, ^96^ supporting the view that the presence of a significant level of parasitemia over a period of time is necessary for the induction and maintenance of AMB cells.

The important question is—can these AMB cells be induced by *Plasmodium* antigens during the infection, and differentiate into antibody‐producing cells and respond to malaria antigens the same way as classical memory B cells? No studies on human AMB have so far directly demonstrated that they differentiate into plasma cells, which secrete antibodies. Single‐cell antibody cloning from circulating AMB obtained from asymptomatic semi‐immune adults showed that AMB B‐cell receptors (BCR) codified for *P. falciparum* ‐neutralizing antibodies, suggesting these cells could potentially contribute to the pool of *P. falciparum*‐ neutralizing antibodies detected in serum and play a protective role.^97^ High levels of secretory IgG transcripts from cloned AMB that match amino acid sequences of antibodies found in circulation support the idea that these cells secrete antibodies in vivo.^97^ However, in vitro restimulation of sorted AMB suggests otherwise; there is reduced Ca^2+^ mobilization, proliferation, cytokine production, and antibody secretion in response to BCR cross‐linking.^24^, ^26^ This has led to the hypothesis that AMB might represent an exhausted/dysfunctional/anergic B cell subset, which might not allow an effective antibody response to the parasite to develop and thus promoting chronic infection. However*,* in vitro assays to determine B‐cell capacity have relied on culture conditions developed for classical memory B cells. FCRL4^+^CD27^‐^CD21^‐^ AMB, contrary to classical memory B cells, proliferate poorly in response to either BCR ligation or *Staphylococcus aureus* stimulation, but can secrete high levels of immunoglobulins in response to IL‐2 and IL‐10 or to IL‐2, IL‐10, and CD40L.^90^ Thus, activation requirements for AMB might be different from those required by classical memory B cells. In addition, the high expression of activation, proliferation, and apoptosis markers on AMB^24^, ^49^, ^97^ might render these cells more sensitive to the manipulation required for cell sorting and in vitro restimulation.

The majority of the studies of AMB in human malaria have been performed on peripheral blood B cells, as the only accessible human lymphoid tissue. B cells and other immune cells normally initiate their response to antigen in lymphoid organs, whereas the blood contains mostly cells either circulating or trafficking to lymphoid organs, and it is not known how far peripheral blood represents B‐cell responses in lymphoid organs. In addition, the frequency of B cells in blood specific to any given antigen is very low, which makes difficult the investigation of their functional and developmental capacity. Therefore, we used our mouse model of mosquito‐transmitted *P. chabaudi* malaria to investigate *Plasmodium*‐specific memory B cells. Using a transgenic mouse strain with high frequencies of B cells specific to the 21‐kDa C‐terminal fragment of *P. chabaudi* merozoite surface protein 1 (MSP1_21_),^49^ *Plasmodium*‐specific AMB could be tracked. We observed the expansion in the spleen of a distinctive *P. chabaudi*‐specific CD11b^+^CD11c^+^ B‐cell subset expressing similar surface markers as human AMB during the persistent stage of erythrocytic infection. In agreement with an AMB phenotype described in humans, these cells were class‐switched and expressed low levels of CD21 and high levels of mouse FCRL5, CD80, and CD273, the last two previously shown to be expressed on mouse memory B cells. Transcriptome analysis of these cells compared well with previously published data of human AMB transcripts. In agreement with data on human AMB, *P. chabaudi*‐specific CD11b^+^CD11c^+^ B cells expressed high levels of *Tbx21* (T‐bet), *Fcrl5*, *Ifng*, *Pdcd1* (PD1), *Mki67*, and class‐switched immunoglobulins, including *Igha*, *Ighg1*, *Ighg2b*, *Ighg2c*, and *Ighg3*. Moreover, similar to human AMB, *P. chabaudi*‐specific mouse AMB require parasite exposure to persist, as their numbers drop to background levels upon resolution of the persistent infection. *P. chabaudi*‐specific AMB also displayed high levels of the proapoptotic genes *Bad*, *Bax*, *Fas*, and *Fasl*, low level of the anti‐apoptotic gene *Bcl2*, and a transcriptome consistent with active replication and activation.^49^ In parallel, and in agreement with our previous data,^48^ a *P. chabaudi*‐specific classical memory B‐cell pool expressing different combinations of the mouse memory markers CD80, CD273, and CD73 together with high levels of *Bcl2* developed and persisted long after resolution of the infection. Interestingly, mouse FCRL5 was highly expressed on all different subsets of *P. chabaudi*‐specific classical memory B cells, including IgM^+^.^49^ FCRL5 was also highly expressed on classical memory B cells generated through immunizations of mice.^49^, ^98^ Persistent infection therefore seems to drive expansion of *P. chabaudi*‐specific mouse AMB, but their existence does not prevent the generation of *P. chabaudi*‐specific classical memory B cells nor prevent resolution of the infection. These observations support the view that constant immune activation rather than impaired memory function leads to the accumulation of AMB in malaria. Nonetheless, although showing very high levels of IgG transcripts, the contribution of *P. chabaudi*‐specific AMB to the serum antibody pool and to protection from *P. chabaudi* infections remains to be demonstrated.

The occurrence of CD11b^+^CD11c^+^T‐bet^+^ AMB‐like B cells has been extensively described in mouse models of chronic viral and bacterial infections, and in many cases are shown to be a fully functional B cell subset able to contribute to protection from infection.^99^, ^100^, ^101^ The peak of CD11c^+^T‐bet^+^ B‐cell production in the spleen is detected early after *Ehrlichia muris* peak infection, and these cells persist thereafter in lower numbers but above background levels accompanying the persistent infection. These cells very much resemble the *P. chabaudi*‐specific AMB (ie, CD11b^+^CD11c^+^Tbet^+^CD273^+^CD73^+^CD80^+^Fas‐L^hi^), with the exemption of being IgM^+^. Moreover, similar to *P. chabaudi*‐specific AMB, the CD11c^+^T‐bet^+^ B cells expanded in response to *E. muris* resembled plasmablasts, including expression of CD138.^49^, ^101^ In accordance, these cells give rise to antibody‐secreting cells that produced antibodies which recognized *E. muris* antigens.^102^, ^103^ Moreover, AMB‐like cells have also been shown to be the original source of protective virus‐specific antibodies in mice. A CD11b^+^CD11c^+^T‐bet^+^ AMB subset appears at the peak of murine gamma herpesvirus 68 (gHV68), lymphocytic choriomeningitis virus (LCMV), murine cytomegalovirus (MCMV), vaccinia, and Friend virus infections.^100^, ^104^ These cells are required for production of virus‐specific IgG2a and are critical to clear gHV68 infection.^100^, ^104^ A T‐bet^+^ AMB subset is also critical for the production of protective IgG2a and to control chronic LCMV cl13 infection.^99^ Thus, in the strong Th1‐biased context of intracellular bacterial and viral infections, AMB appear to be a source of protective antibodies.

A subset of CD11c^+^T‐bet^+^ AMB‐like cells, originally termed age‐associated B cells, have also been shown to expand in several mouse models of autoimmune disorders as well as in blood of subjects suffering autoimmune diseases such as rheumatoid arthritis, systemic lupus erythematosus, multiple sclerosis, and Crohn's disease.^88^, ^105^, ^106^, ^107^, ^108^, ^109^ In mouse models of lupus‐like disease, AMB‐like cells expressed CD138, characteristic of antibody secreting plasma cells/plasmablasts, and directly contributed to the production of auto‐antibodies.^110^ Moreover, in the context of autoimmune disorders, AMB‐like cells showed potent antigen‐presenting capacity and production of proinflammatory cytokines.^108^, ^110^, ^111^ Similar to malaria, AMB‐like cells identified in autoimmunity showed signs of previous activation and proliferation in vivo, and defective Ca^2+^ signaling and poor proliferation in response to BCR stimulation in vitro.^112^, ^113^ Surprisingly, protective AMB‐like cells generated during *E. muris* infection also produced polyreactive autoantibodies.^102^, ^103^ This is intriguing and surprising, as these autoantibodies seem not to drive pathology in the *E. muris* infection model. A recent study showed that an AMB‐like CD11c^+^T‐bet^+^ B‐cell subset expanded in the spleen during acute blood stage *Plasmodium yoelii* 17XNL infection in mice and secreted autoantibodies specific to erythrocyte phosphatidylserine, which appear to drive anemia.^114^ This opens the intriguing possibility that AMB might contribute to malaria pathology through the production of autoantibodies.

Nonetheless, whether protective or pathogenic, the outstanding majority of data on AMB‐like cells obtained in viral and bacterial infections as well as autoimmune disorders is so far not compatible with the hypothesis that AMB are exhausted or anergic. It might be argued that these AMB‐like subsets generated in different immune scenarios, despite sharing an impressive number of key characteristics, are in fact intrinsically different from the AMB cells observed in malaria. Alternatively, although being the same subset, different immune scenarios might differentially affect their function, with malaria and HIV preferentially driving AMB to exhaustion unlike other Th‐1‐biased chronic infections such as LCMV. If the latter is the case, then those signals affecting AMB which are unique to malaria and not shared by other chronic infections or autoimmune disorders remain to be identified.

Together, most of these studies in human and experimental malaria as well as other infections suggest that AMB represent an effector B‐cell subset generated in, and sustained by, persistent antigen exposure. However, there are reports of relatively long AMB persistence in the apparent absence of malaria exposure,^68^ and long‐lived T‐bet^+^ memory B cells have been described in the *E. muris* infection model.^115^ Therefore, AMB in malaria and beyond represent an exciting field of study with many unanswered questions. What is the true nature of AMB? Do they contribute to protection, pathology, or both? Are they dysfunctional/exhausted/anergic? Do they contribute to the memory pool*?*

### 1.3 What are the signals required to generate long‐lasting B‐cell responses to *plasmodium*?

Although there is evidence of T‐independent B1 B cells, which bridge innate and adaptive responses, producing memory responses (B1 cells defined in mice by expression of IgM^hi^IgD^lo^CD23^−^B220^lo^, and in humans by CD20^+^CD27^+^CD43^+^CD38^lo/int^), it is the adaptive response of B2 B cells (in mice B220^+^AA4.1^−^CD23^+^IgM^int^IgD^hi^, and humans CD20^+^CD27^+^CD43^−^ B cells), and GC interactions which are critical for long‐lasting B‐cell responses in the majority of memory responses.^31^, ^116^, ^117^ Despite the importance of antibody responses in malaria, details on GC formation and activation/regulation of T cell help by follicular helper T‐cell in malaria are only starting to emerge. CD4^+^ T cells producing IL‐21, a characteristic cytokine of Tfh, and Tfh defined by expression of PD‐1 and CXCR5 are present in peripheral blood mononuclear cells from malaria‐exposed immune adults^118^, ^119^, ^120^ and in some cases, correlate with *P falciparum*‐specific IgG antibodies in children with acute *P. falciparum* malaria.^121^ Alterations in the GC B‐cell response to *Plasmodium* infection will very likely affect generation of long‐lasting B cell memory, but this is difficult to investigate fully using human peripheral blood cells, and we need to turn to experimental models to elucidate GC interactions in malaria. *Plasmodium* infections in mice trigger a robust GC response, and CD4^+^ T cells producing IL‐21, of which a substantial proportion have a Tfh cell phenotype, are required to generate GC B‐cell and IgG responses, and to resolve *P. chabaudi* and *P. yoelii* infections.^10^, ^50^, ^122^, ^123^, ^124^ Moreover, mice deficient in IL‐21 signaling are not immune to reinfection, and fail to generate *P. chabaudi*‐specific long‐lived plasma cells and memory B cells.^10^ *P. chabaudi*‐specific IgM responses are not altered in the absence of IL‐21 signaling, thus showing that IgM is not sufficient to clear the infection,^10^ and that IL‐21 is essential to generate long‐lasting class‐switched protective B cell responses. Although IL‐21 is not required to activate Tfh cells in *P. chabaudi* infections, Tfh cells are an important source of IL‐21 and are also essential to generate class‐switched antibody responses and clear the infection.^10^, ^122^

The pattern recognition receptor cyclic GMP‐AMP synthase (cGAS), ICOS, and IL‐10 signaling on B cells are all required for GC B‐cell and IgG responses,^125^ and this is the case also in experimental *P. yoelii* infections.^123^, ^126^, ^127^ Thus, these signals are probably also essential for generation of *Plasmodium*‐specific memory B cells. The signaling lymphocytic activation molecule (SLAM)‐associated protein (SAP), shown to be necessary for GC and B‐cell memory responses to immunizations and viral infections,^128^, ^129^ has only a partial impact on IgG and GC responses in *P. chabaudi* infections, but SAP does contribute to some control of chronic infection.^122^ A requirement for SAP interactions in immunity to reinfection in *Plasmodium* remains unexplored.

Although GC formation clearly takes place in experimental malaria, there is some indication that this may not be optimal as GC responses are enhanced by in vivo blockade of CTLA‐4, or PD‐L1 in combination with LAG‐3.^130^, ^131^ However, another interpretation is that GC formation during *Plasmodium* infection is normal, and that the blockade is simply overriding the normal mechanism of control of GC and B‐cell responses. Malaria is characterized by a strong Th1–like response, which affect B cell responses. The signature Th1 cytokine, IFN‐γ, is responsible for switching to IgG2a/c antibodies in mice, and for the human analogues IgG1 and IgG3,^132^, ^133^, ^134^ isotypes which activate complement and Fcγ receptors on macrophages bringing about pathogen killing and phagocytosis. These isotypes have been shown to clear viral infections,^135^, ^136^ and to correlate with protection against *Plasmodium* infections.^137^, ^138^

There are, however, seemingly opposing views on whether Th1 responses promote or impair B cells and GCs in experimental *Plasmodium* infections. On ‐one hand, *Plasmodiu*m‐specific Th1 CD4 T cells can support B‐cell responses.^137^, ^138^, ^139^ On the other hand, IFN‐γ and TNF‐α responses reduce activation of Tfh and GC B‐cell responses in *Plasmodium berghei* infections and *P. yoelii* infections in mice.^124^, ^126^, ^140^, ^141^ Similarly, a Th1‐polarized Tfh subset found expanded in blood from infected children shows reduced capacity to support B cell activation in vitro.^119^ These data suggest that while Th1 signaling is important for switching to potentially protective antibody isotypes, in excess it might also negatively limit normal development of the GC response. As yet, the direct impact of these signals on the longevity of memory B‐cell responses has not been demonstrated. However, and similar to viral and intracellular bacterial infections, acute *P. chabaudi* blood‐stage infection triggers a robust Th1 response,^10^ and yet we detect substantial GC formation and a long‐lived B cell and antibody responses, suggesting that IFN‐γ production by Th1 cells is not sufficient per se to prevent robust humoral responses.

Another contribution of Th1 responses and IFN‐γ to the B‐cell response is their role in the generation of AMB cells. Indeed, T‐bet expression in B cells in malaria is regulated by IFN‐γ,^92^ triggered by a combination of CD40, IL‐12, and IL‐18 signaling. AMB cells are not activated by cross‐linking the BCR unlike classical memory B cells, which may explain why they are difficult to activate in vitro, but age‐associated CD11c^+^T‐bet^+^ B cells can be activated by TLR 7 and 9 signaling,^87^, ^110^ and antigen‐specific AMB‐like B cells in mice are transiently expanded after immunization with a TLR7/8 ligand together with Hen Egg Lysosome^100^ or a recombinant *Plasmodium* antigen.^49^ IFN‐γ or IL‐21 together with TLR signaling drives the generation of CD11c^+^T‐bet^+^ AMB‐like cells,^142^ which is particularly relevant in *Plasmodium* infections where there is a large co‐expression of IFN‐γ and IL‐21 by CD4^+^ T cells.^10^ Similarly, IL‐21 is required to activate AMB‐like cells in response to *E. muris* infection,^143^ and IL‐21 and SAP are required to activate AMB‐like cells during autoimmunity.^144^ These data support the view that the signals to activate AMB in a variety of situations including malaria where there is or has been chronic antigen presence, are partially but not entirely shared with those required for the generation of classical memory B cells.

**Figure 3:**
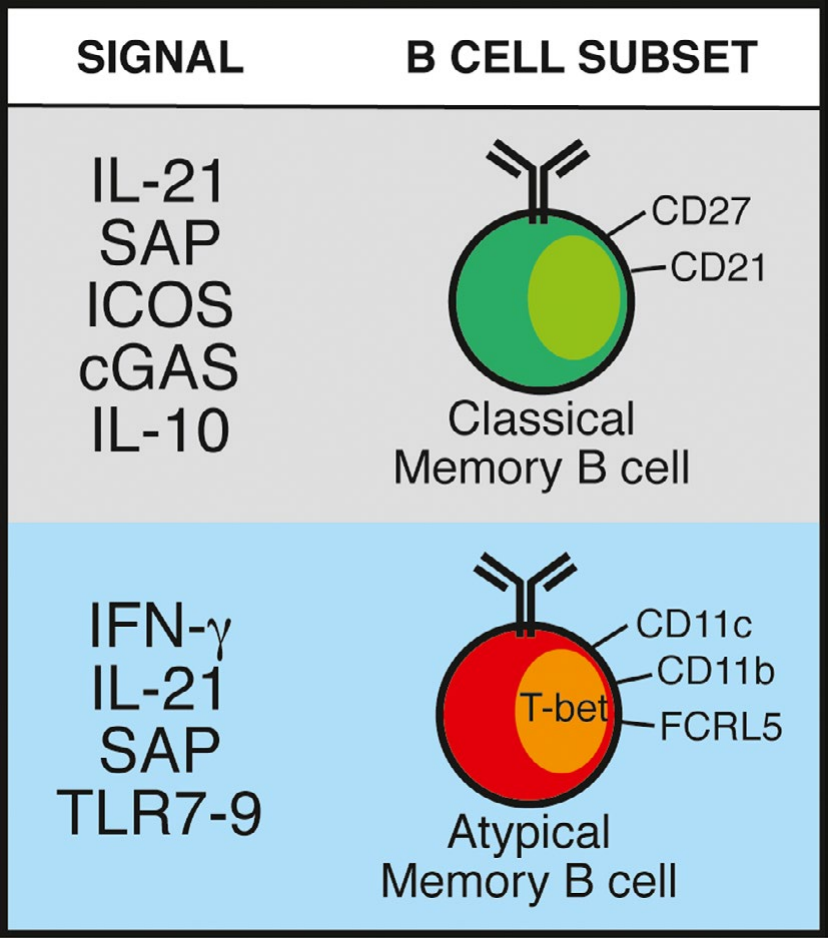
Signals known or suggested to support the generation of either classical or atypical memory B cells in response to *Plasmodium* spp. infection. SAP: signaling lymphocyte activation molecule (SLAM)‐associated protein; ICOS: inducible T cell co‐stimulator; cGAS: pattern recognition receptor cyclic GMP‐AMP synthase; TLR: toll‐like receptor

We are left with apparently contradictory findings. The same signals that seem to impair GC responses during malaria promote generation of AMB‐like cells. Are AMB cells a GC subset or are they generated outside of the GC, and is T cell support required for their generation? Most AMB cells have surface IgG, and Immunoglobulin variable regions from AMB generated during malaria are heavily mutated, suggesting a GC origin.^24^, ^97^, ^145^ Signals able to drive AMB‐like cell activation, including IL‐21 and SAP, seem to also suggest a GC origin, and MHCII has been shown to be required to activate AMB like cells to *E. muris.* 143, 144 T‐bet^+^ AMB‐like cells have been suggested to be involved in the formation of spontaneous GC in a mouse model of lupus.^146^ Moreover, extrafollicular development of T‐bet‐expressing plasmablasts has been reported in response to *E. muris* infection,^102^, ^143^ suggesting that AMB‐like cells might also arise independently of the GC. To date, the origin of AMB during malaria remains uncertain. If, as some studies suggest, AMB and classical memory B cells share a common developmental origin,^24^, ^145^ then a key element might be the balance between IL‐21 and IFN‐γ, with excessive IFN‐γ favoring GC disruption and AMB accumulation, while IL‐21 supporting both AMB and classical memory B cell generation. However, it is important to highlight that the robust IFN‐γ responses during *P. chabaudi* and other intracellular viral and bacterial pathogens do not preclude protective long‐lasting B‐cell and antibody responses, nor disrupt the GC response. In addition, the role of IL‐21 and GC in the generation of AMB in response to *Plasmodium* infection remains unexplored. Figure 3 summarizes the main signals, either known or predicted, to be involved in the generation of classical and AMB.

## 2 CONCLUDING REMARKS

Many studies show that B‐cell and antibody responses to malaria are short‐lived and often low level or absent, particularly in young children in endemic areas. Given the very varied methods of B cell or antibody measurement in a large variety of studies, the different transmission intensities of the study sites, and in some cases single time‐point measurements at unknown times after a detectable parasitemia, it is hard to put together a cohesive picture of what is happening. Low levels of antibodies and/or drops in antibody titer may be due in part to their production by short‐lived plasma cells in acute infection (Figure 1B), and we do not know when in the kinetics of the humoral response antibody measurements have been taken. Thus, the drop in antibody titer may well represent the contraction phase of a normal B‐cell response rather than evidence of a deficient B cell memory response to *Plasmodium*. However, some studies clearly show poor *Plasmodium*‐specific antibody responses in endemic areas and we need to understand the reasons for this. It is difficult to carry out a systematic study over time in children or adults in all field settings following a defined infection. Controlled human *Plasmodium* infections (CHMI) of adults in endemic countries, only recently made possible, may help us elucidate this important aspect of the humoral response in malaria.

There is, conversely, a large body of literature showing long‐lived B‐cell and antibody responses to *Plasmodium* parasites in humans and animal models, which challenge the concept of *Plasmodium* infection always driving the B‐cell response to exhaustion of dysfunction. Studies being performed in areas of different transmission intensity with consequently different reinfection rates might explain some of the apparent discrepancies. AMB, suggested as a reason for dysfunctional B‐cell responses to malaria, may not be the culprit. Whether they could be a source of protective antibodies and thus play a role in immunity to *Plasmodium* as has been shown in other infections in experimental models, remains to be conclusively demonstrated. The restimulation protocols used to demonstrate AMB anergy during malaria have not been exhaustive and might not be adequate to stimulate this subset of B cells. Moreover, even if AMB detected in the blood of malaria exposed individuals were indeed anergic, this would not rule out the possibility these might be terminally differentiated B‐cell subsets which have contributed to an antibody response in lymphoid organs during their past history. Thus, the true nature of AMB in malaria still remains elusive and intriguing, and animal models will very likely shed light on their origins, developmental history, and function during infection. Understanding the mechanisms that govern differential longevity of protective B‐cell responses among different individuals or to different *Plasmodium* antigens will greatly expand our capacity to improve antibody‐based malaria vaccines. In this regard, it is important to keep in mind that a single B cell target might not be sufficient to confer protection from complex protozoan parasites, including *Plasmodium*. Putative strategies might combine vaccines against different targets which provide protection by different mechanisms and target different stages of the parasite life cycle, while taking into account the occurrence of polymorphisms on the selected targets. Moreover, B‐cell responses might not be sufficient, and modulation of other immune arms might be required to confer full protection from *Plasmodium* infection while preventing pathology.

## References

[1] World Health Organization . World malaria report 2018. Geneva, Switzerland: World Health Organization; 2018 1‐210.

[2] Alonso PL , Sacarlal J , Aponte JJ , et al. Efficacy of the RTS, S/AS02A vaccine against *Plasmodium falciparum* infection and disease in young African children: Randomised controlled trial. Lancet. 2004;364:1411‐1420. 15488216 10.1016/S0140-6736(04)17223-1

[3] Crotty S . Follicular helper CD4 T cells (TFH). Annu Rev Immunol. 2011;29:621‐663. 21314428 10.1146/annurev-immunol-031210-101400

[4] Fowkes FJI , Richards JS , Simpson JA , Beeson JG . The relationship between anti‐merozoite antibodies and incidence of *Plasmodium falciparum* malaria: A systematic review and meta‐analysis. PLoS Medicine. 2010;7:e1000218. 20098724 10.1371/journal.pmed.1000218 PMC2808214

[5] Cohen S , McGREGOR IA , Carrington S . Gamma‐globulin and acquired immunity to human malaria. Nature. 1961;192:733‐737. 13880318 10.1038/192733a0

[6] Sabchareon A , Burnouf T , Ouattara D , et al. Parasitologic and clinical human response to immunoglobulin administration in falciparum malaria. Am J Trop Med Hyg. 1991;45(3):297-308. 1928564 10.4269/ajtmh.1991.45.297

[7] Osier FHA , Fegan G , Polley SD , et al. Breadth and magnitude of antibody responses to multiple *Plasmodium falciparum* merozoite antigens are associated with protection from clinical malaria. Infect Immun. 2008;76:2240‐2248. 18316390 10.1128/IAI.01585-07 PMC2346713

[8] Burns JM , Dunn PD , Russo DM . Protective immunity against *Plasmodium yoelii* malaria induced by immunization with particulate blood‐stage antigens. Infect Immun. 1997;65:3138‐3145. 9234766 10.1128/iai.65.8.3138-3145.1997 PMC175443

[9] von der Weid T , Langhorne J , Honarvar N . Gene‐targeted mice lacking B cells are unable to eliminate a blood stage malaria infection. J Immunol. 1996;156:2510‐2516. 8786312

[10] Pérez‐Mazliah D , Ng DHL , Freitas do Rosário AP , et al. Disruption of IL‐21 signaling affects T cell‐B cell interactions and abrogates protective humoral immunity to malaria. PLoS Pathog. 2015;11:e1004715. 25763578 10.1371/journal.ppat.1004715 PMC4370355

[11] Hill DL , Schofield L , Wilson DW . IgG opsonization of merozoites: Multiple immune mechanisms for malaria vaccine development. Int J Parasitol. 2017;47:585‐595. 28668325 10.1016/j.ijpara.2017.05.004

[12] Teo A , Feng G , Brown GV , Beeson JG , Rogerson SJ . Functional antibodies and protection against blood‐stage malaria. Trends Parasitol. 2016;32:887‐898. 27546781 10.1016/j.pt.2016.07.003

[13] Chan J‐A , Howell KB , Reiling L , et al. Targets of antibodies against *Plasmodium falciparum*–infected erythrocytes in malaria immunity. J Clin Invest. 2012;122:3227‐3238. 22850879 10.1172/JCI62182 PMC3428085

[14] Clark IA , Budd AC , Alleva LM , Cowden WB . Human malarial disease: A consequence of inflammatory cytokine release. Malar J. 2006;5:1‐32. 17029647 10.1186/1475-2875-5-85 PMC1629020

[15] Donati D , Zhang LP , Chen Q , et al. Identification of a polyclonal B‐Cell activator in *Plasmodium falciparum* . Infect Immun. 2004;72:5412‐5418. 15322039 10.1128/IAI.72.9.5412-5418.2004 PMC517431

[16] Rosenberg YJ . Autoimmume and polyclonal B cell responses during murine malaria. Nature. 1978;274:170‐172. 351426 10.1038/274170a0

[17] Abele DC , Tobie JE , Hill GJ , Contacos PG , Evans CB . Alterations in serum proteins and 19S antibody production during the course of induced malarial infections in man. Am J Trop Med Hyg. 1965;14:191‐197. 14270444 10.4269/ajtmh.1965.14.191

[18] Cadman ET , Abdallah AY , Voisine C , et al. Alterations of splenic architecture in malaria are induced independently of Toll‐like receptors 2, 4, and 9 or MyD88 and may affect antibody affinity. Infect Immun. 2008;76:3924‐3931. 18559428 10.1128/IAI.00372-08 PMC2519400

[19] Bockstal V , Geurts N , Magez S . Acute disruption of bone marrow B Lymphopoiesis and apoptosis of transitional and marginal zone B Cells in the spleen following a blood‐stage *Plasmodium chabaudi* infection in mice. J Parasitol Res. 2011;2011:1-11. 10.1155/2011/534697 PMC3112522 21687602

[20] Urban BC , Hien TT , Day NP , et al. Fatal *Plasmodium falciparum* malaria causes specific patterns of splenic architectural disorganization. Infect Immun. 2005;73:1986‐1994. 15784539 10.1128/IAI.73.4.1986-1994.2005 PMC1087405

[21] Belyaev NN , Brown DE , Diaz A‐IG , et al. Induction of an IL7‐R + c‐Kit hi myelolymphoid progenitor critically dependent on IFN‐γ signaling during acute malaria. Nat Immunol. 2010;11:477‐485. 20431620 10.1038/ni.1869

[22] Belyaev NN , Biró J , Langhorne J , Potocnik AJ . Extramedullary myelopoiesis in Malaria depends on mobilization of myeloid‐restricted progenitors by IFN‐γ induced chemokines. PLoS Pathog. 2013;9:e1003406. 23762028 10.1371/journal.ppat.1003406 PMC3675198

[23] Weiss GE , Crompton PD , Li S , et al. Atypical memory B cells are greatly expanded in individuals living in a malaria‐endemic area. J Immunol. 2009;183:2176‐2182. 19592645 10.4049/jimmunol.0901297 PMC2713793

[24] Portugal S , Tipton CM , Sohn H , et al. Malaria‐associated atypical memory B cells exhibit markedly reduced B cell receptor signaling and effector function. Elife. 2015;4:e07218. 10.7554/eLife.07218 PMC4444601 25955968

[25] Illingworth J , Butler NS , Roetynck S , et al. Chronic exposure to *Plasmodium falciparum* is associated with phenotypic evidence of B and T cell exhaustion. J Immunol. 2013;190:1038‐1047. 23264654 10.4049/jimmunol.1202438 PMC3549224

[26] Moir S , Ho J , Malaspina A , et al. Evidence for HIV‐associated B cell exhaustion in a dysfunctional memory B cell compartment in HIV‐infected viremic individuals. J Exp Med. 2008;205(8):1797-1805. 18625747 10.1084/jem.20072683 PMC2525604

[27] Oliviero B , Mantovani S , Ludovisi S , et al. Skewed B cells in chronic hepatitis C virus infection maintain their ability to respond to virus‐induced activation. J Viral Hepat. 2015;22:391‐398. 25258145 10.1111/jvh.12336

[28] Joosten SA , van Meijgaarden KE , del Nonno F , et al. Patients with tuberculosis have a dysfunctional circulating B‐cell compartment, which normalizes following successful treatment. PLoS Pathog. 2016;12:e1005687. 27304615 10.1371/journal.ppat.1005687 PMC4909319

[29] Rubtsov AV , Marrack P , Rubtsova K . T‐bet expressing B cells – Novel target for autoimmune therapies? Cell Immunol. 2017;321:35‐39. 28641866 10.1016/j.cellimm.2017.04.011

[30] Rubtsova K , Rubtsov AV , Cancro MP , Marrack P . Age‐associated B cells: A T‐bet? dependent effector with roles in protective and pathogenic immunity. J Immunol. 2015;195:1933‐1937. 26297793 10.4049/jimmunol.1501209 PMC4548292

[31] Kurosaki T , Kometani K , Ise W . Memory B cells. Nat Rev Immunol. 2015;15:149‐159. 25677494 10.1038/nri3802

[32] Amanna IJ , Carlson NE , Slifka MK . Duration of humoral immunity to common viral and vaccine antigens. N Engl J Med. 2007;357:1903‐1915. 17989383 10.1056/NEJMoa066092

[33] Yu X , Tsibane T , McGraw PA , et al. Neutralizing antibodies derived from the B cells of 1918 influenza pandemic survivors. Nature. 2008;455:532‐536. 18716625 10.1038/nature07231 PMC2848880

[34] Slifka MK , Ahmed R . Limiting dilution analysis of virus‐specific memory B cells by an ELISpot assay. J Immunol Methods. 1996;199:37‐46. 8960096 10.1016/s0022-1759(96)00146-9

[35] Hammarlund E , Thomas A , Poore EA , et al. Durability of vaccine‐induced immunity against tetanus and diphtheria toxins: A cross‐sectional analysis. Clin Infect Dis. 2016;62:1111‐1118. 27060790 10.1093/cid/ciw066 PMC4826453

[36] Hammarlund E , Lewis MW , Hansen SG , et al. Duration of antiviral immunity after smallpox vaccination. Nat Med. 2003;9:1131‐1137. 12925846 10.1038/nm917

[37] Crotty S , Felgner P , Davies H , Glidewell J , Villarreal L , Ahmed R . Cutting edge: Long‐term B cell memory in humans after smallpox vaccination. J Immunol. 2003;171:4969‐4973. 14607890 10.4049/jimmunol.171.10.4969

[38] Maple PAC , Jones CS , Wall EC , et al. Immunity to diphtheria and tetanus in England and Wales. Vaccine. 2000;19:167‐173. 10930669 10.1016/s0264-410x(00)00184-5

[39] Ly A , Hansen DS . Development of B cell memory in malaria. Front Immunol. 2019;10:111‐137. 31001244 10.3389/fimmu.2019.00559 PMC6454213

[40] Cockburn IA , Seder RA . Malaria prevention: from immunological concepts to effective vaccines and protective antibodies. Nat Immunol. 2018;19:1199‐1211. 30333613 10.1038/s41590-018-0228-6

[41] EL Silveira, V , Dominguez MR , Soares IS . To B or not to B: Understanding B Cell responses in the development of malaria infection. Front Immunol. 2018;9:1‐9. 30619319 10.3389/fimmu.2018.02961 PMC6302011

[42] Marsh K , Otoo L , Hayes RJ , Carson DC , Greenwood BM . Antibodies to blood stage antigens of *Plasmodium falciparum* in rural Gambians and their relation to protection against infection. Trans R Soc Trop Med Hyg. 1989;83:293‐303. 2694458 10.1016/0035-9203(89)90478-1

[43] Gupta S , Snow RW , Donnelly CA , Marsh K , Newbold C . Immunity to non‐cerebral severe malaria is acquired after one or two infections. Nat Med. 1999;5:340. 10086393 10.1038/6560

[44] Kinyanjui SM , Conway DJ , Lanar DE , Marsh K . IgG antibody responses to *Plasmodium falciparum* merozoite antigens in Kenyan children have a short half‐life. Malar J. 2007;6:1318‐1319. 10.1186/1475-2875-6-82 PMC1920526 17598897

[45] Akpogheneta OJ , Duah NO , Tetteh KKA , et al. Duration of naturally acquired antibody responses to blood‐stage *Plasmodium falciparum* is age dependent and antigen specific. Infect Immun. 2008;76:1748‐1755. 18212081 10.1128/IAI.01333-07 PMC2292892

[46] Weiss GE , Traore B , Kayentao K , et al. The *Plasmodium falciparum*‐specific human memory B cell compartment expands gradually with repeated malaria infections. PLoS Pathog. 2010;6:e1000912. 20502681 10.1371/journal.ppat.1000912 PMC2873912

[47] Crompton PD , Kayala MA , Traore B , et al. A prospective analysis of the Ab response to *Plasmodium falciparum* before and after a malaria season by protein microarray. Proc Natl Acad Sci. 2010;107:6958‐6963. 20351286 10.1073/pnas.1001323107 PMC2872454

[48] Ndungu FM , Cadman ET , Coulcher J , et al. Functional memory B cells and long‐lived plasma cells are generated after a single *Plasmodium chabaudi* infection in mice. PLoS Pathog. 2009;5:e1000690. 20011127 10.1371/journal.ppat.1000690 PMC2784955

[49] Pérez‐Mazliah D , Gardner PJ , Schweighoffer E , et al. *Plasmodium*‐specific atypical memory B cells are short‐lived activated B cells. Elife. 2018;7:e39800. 30387712 10.7554/eLife.39800 PMC6242553

[50] Achtman AH , Stephens R , Cadman ET , Harrison V , Langhorne J . Malaria‐specific antibody responses and parasite persistence after infection of mice with *Plasmodium chabaudi chabaudi* . Parasite Immunol. 2007;29:435‐444. 17727567 10.1111/j.1365-3024.2007.00960.x

[51] Chougnet C , Deloron P , Lepers JP , et al. Humoral and cell‐mediated immune responses to the *Plasmodium falciparum* Antigens PF155/RESA and CS protein: Seasonal variations in a population recently reexposed to endemic malaria. Am J Trop Med Hyg. 1990;43:234‐242. 2221217 10.4269/ajtmh.1990.43.234

[52] Migot F , Chougnet C , Raharimalala L , Astagneau P , Lepers JP , Deloron P . Human immune responses to the *Plasmodium falciparum* ring‐infected erythrocyte surface antigen (Pf155/RESA) after a decrease in malaria transmission in Madagascar. Am J Trop Med Hyg. 1993;48:432‐439. 8470778 10.4269/ajtmh.1993.48.432

[53] Migot F , Chougnet C , Henzel D . et al. Anti‐malaria antibody‐producing B cell frequencies in adults after a *Plasmodium falciparum* outbreak in Madagascar. Clin Exp Immunol. 1995;102:529‐534. 8536368 10.1111/j.1365-2249.1995.tb03848.x PMC1553358

[54] Waa JAV , Jensen JB , Akood MA , Bayoumi R . Longitudinal study on the in vitro immune response to *Plasmodium falciparum* in Sudan. Infect Immun. 1984;45:505‐510. 6378799 10.1128/iai.45.2.505-510.1984 PMC263274

[55] Moncunill G , Mayor A , Jiménez A , et al. High antibody responses against *Plasmodium falciparum* in immigrants after extended periods of interrupted exposure to malaria. PLoS ONE. 2013;8:1‐11. 10.1371/journal.pone.0073624 PMC3743903 23967347

[56] Yman V , White MT , Asghar M , et al. Antibody responses to merozoite antigens after natural *Plasmodium falciparum* infection: Kinetics and longevity in absence of re‐exposure. BMC Med. 2019;1‐14. 30696449 10.1186/s12916-019-1255-3 PMC6352425

[57] Jelinek T , Schulte C , Infectious RBC . Imported *falciparum* malaria in Europe: Sentinel surveillance data from the European network on surveillance of imported infectious diseases. Clin Infect Dis. 2002;2002(34):572‐576. 10.1086/338235 11803507

[58] Matteelli A , Colombini P , Gulletta M , Castelli F , Carosi G . Epidemiological features and case management practices of imported malaria in northern Italy 1991–1995. Trop Med Int.Heal. 1999;4:653‐657. 10.1046/j.1365-3156.1999.00468.x 10583898

[59] Perri GD , Solbiati M , Vento S ,. West African immigrants and new patterns of malaria imported to North Eastern Italy. J Travel Med. 1994;1994(1):147‐151. 10.1111/j.1708-8305.1994.tb00582.x 9815329

[60] do Rosário AP , Langhorne, J . T cell‐derived IL‐10 and its impact on the regulation of host responses during malaria. Int J Parasitol. 2012;42:549‐555. 22549022 10.1016/j.ijpara.2012.03.010

[61] Ndungu FM , Lundblom K , Rono J , Illingworth J , Eriksson S , Färnert A . Long‐lived *Plasmodium falciparum* specific memory B cells in naturally exposed Swedish travelers. Eur J Immunol. 2013;43:2919‐2929. 23881859 10.1002/eji.201343630 PMC4114544

[62] Chougnet C , Deloron P , Savel J . Persistence of cellular and humoral response to synthetic peptides from defined *Plasmodium falciparum* antigens. Ann Trop Med Parasitol. 2016;85:357‐363. 10.1080/00034983.1991.11812574 1720947

[63] Braga EM , Fontes CJF , Krettli AU . Persistence of humoral response against sporozoite and blood‐stage malaria antigens 7 years after a brief exposure to *Plasmodium vivax* . J Infect Dis. 1998; 177:1132‐1135. 9535000 10.1086/517412

[64] Wipasa J , Suphavilai C , Okell LC , et al. Long‐lived antibody and B cell memory responses to the human malaria parasites, *Plasmodium falciparum* and *Plasmodium vivax* . PLoS Pathog. 2010;6:e1000770. 20174609 10.1371/journal.ppat.1000770 PMC2824751

[65] Nogaro SI , Hafalla JC , Walther B , et al. The breadth, but not the magnitude, of circulating memory B cell responses to *Plasmodium falciparum* increases with age/exposure in an area of low transmission. PLoS ONE. 2011;6(10):e25582. 21991321 10.1371/journal.pone.0025582 PMC3186790

[66] Triller G , Scally SW , Costa G , et al. Natural parasite exposure induces protective human anti‐malarial antibodies. Immunity. 2017;47:1197‐1209e10. 29195810 10.1016/j.immuni.2017.11.007 PMC5738265

[67] Murugan R , Buchauer L , Triller G , et al. Clonal selection drives protective memory B cell responses in controlled human malaria infection. Sci Immunol. 2018;3:eaap8029. 29453292 10.1126/sciimmunol.aap8029

[68] Changrob S , McHenry AM , Nyunt MH , et al. Persistence of long‐lived memory B cells specific to duffy binding protein in individuals exposed to *Plasmodium vivax* . Sci Rep. 2018;8:1‐11. 29844379 10.1038/s41598-018-26677-x PMC5973932

[69] Taylor RR , Egan A , McGuinness D ,et al. Selective recognition of malaria antigens by human serum antibodies is not genetically determined but demonstrates some features of clonal imprinting. Int Immunol. 1996;8:905‐915. 8671680 10.1093/intimm/8.6.905

[70] Drakeley CJ , Corran PH , Coleman PG , et al. Estimating medium‐ and long‐term trends in malaria transmission by using serological markers of malaria exposure. Proc Natl Acad Sci. 2005;102:5108‐5113. 15792998 10.1073/pnas.0408725102 PMC555970

[71] Udhayakumar V , Kariuki S , Kolczack M , et al. Longitudinal study of natural immune responses to the *Plasmodium falciparum* apical membrane antigen (AMA‐1) in a holoendemic region of malaria in western Kenya: Asembo Bay Cohort Project VIII. Am J Trop Med Hyg. 2001;65:100‐107. 11508382 10.4269/ajtmh.2001.65.100

[72] Longley RJ , Reyes‐Sandoval A , Montoya‐Díaz E , et al. Acquisition and longevity of antibodies to *Plasmodium vivax* preerythrocytic antigens in Western Thailand. Clin Vaccine Immunol. 2016;23:117‐124. 26656115 10.1128/CVI.00501-15 PMC4744911

[73] Dorfman JR , Bejon P , Ndungu FM , et al. B cell memory to 3 *Plasmodium falciparum* blood‐stage antigens in a malaria‐endemic area. J Infect Dis. 2005;191:1623‐1630. 15838788 10.1086/429671

[74] Ayieko C , Maue AC , Jura WGZO , et al. Changes in B cell populations and merozoite surface protein‐1‐specific memory B cell responses after prolonged absence of detectable *Plasmodium falciparum* infection. PLoS ONE. 2013;8:e67230‐e67312. 23826242 10.1371/journal.pone.0067230 PMC3695086

[75] Ndungu FM , Olotu A , Mwacharo J , et al. Memory B cells are a more reliable archive for historical antimalarial responses than plasma antibodies in no‐longer exposed children. Proc Natl Acad Sci. 2012;109:8247‐8252. 22566630 10.1073/pnas.1200472109 PMC3361387

[76] Guan Y , Sajadi MM , Kamin‐Lewis R , et al. Discordant memory B cell and circulating anti‐env antibody responses in HIV‐1 infection. Proc Natl Acad Sci. 2009;106:3952‐3957. 19225108 10.1073/pnas.0813392106 PMC2644653

[77] Radbruch A , Muehlinghaus G , Luger EO , et al. Competence and competition: The challenge of becoming a long‐lived plasma cell. Nat Publ Gr. 2006;6:741‐750. 10.1038/nri1886 16977339

[78] Ng DHL , Skehel JJ , Kassiotis G , Langhorne J . Recovery of an antiviral antibody response following attrition caused by unrelated infection. PLoS Pathog. 2014;10:e1003843. 24391499 10.1371/journal.ppat.1003843 PMC3879355

[79] Matar CG , Anthony NR , O'Flaherty BM , et al. Gammaherpesvirus co‐infection with malaria suppresses anti‐parasitic humoral immunity. PLoS Pathog. 2015;11:e1004858. 25996913 10.1371/journal.ppat.1004858 PMC4440701

[80] White MT , Griffin JT , Akpogheneta O , et al. Dynamics of the antibody response to *Plasmodium falciparum* infection in African children. J Infect Dis. 2014;210:1115‐1122. 24719471 10.1093/infdis/jiu219

[81] Clark EH , Silva CJ , Weiss GE , et al. *Plasmodium falciparum* malaria in the Peruvian Amazon, a region of low transmission, is associated with immunologic memory. Infect Immun. 2012;80:1583‐1592. 22252876 10.1128/IAI.05961-11 PMC3318420

[82] Deloron P , Chougnet C . Is Immunity to malaria really short‐lived? Parasitol Today. 1992;8:375‐378. 15463545 10.1016/0169-4758(92)90174-z

[83] Bouchaud O , Cot M , Kony S , et al. Do African immigrants living in France have long‐term malarial immunity?. Am J Trop Med Hyg. 2005;72:21‐25. 15728861

[84] Knox JJ , Buggert M , Kardava L , et al. T‐bet+ B cells are induced by human viral infections and dominate the HIV gp140 response. JCI insight. 2017;2:92943. 28422752 10.1172/jci.insight.92943 PMC5396521

[85] Johrens K , Shimizu Y , Anagnostopoulos I , et al. T‐bet‐positive and IRTA1‐positive monocytoid B cells differ from marginal zone B cells and epithelial‐associated B cells in their antigen profile and topographical distribution. Haematologica. 2005;90:1070‐1077. 16079106

[86] Rodrigues V , Laforge M , Campillo‐Gimenez L , et al. Abortive T follicular helper development is associated with a defective humoral response in *Leishmania infantum*‐infected macaques. PLoS Pathog. 2014;10:e1004096‐e1004117. 24763747 10.1371/journal.ppat.1004096 PMC4005728

[87] Hao Y , O'Neill P , Naradikian MS , Scholz JL , Cancro MP . A B‐cell subset uniquely responsive to innate stimuli accumulates in aged mice. Blood. 2011;118:1294‐1304. 21562046 10.1182/blood-2011-01-330530 PMC3152496

[88] Rubtsova K , Rubtsov AV , Thurman JM , Mennona JM , Kappler JW , Marrack P . B cells expressing the transcription factor T‐bet drive lupus‐like autoimmunity. J Clin Invest. 2017;127:1392‐1404. 28240602 10.1172/JCI91250 PMC5373868

[89] Sullivan RT , Kim CC , Fontana MF , et al. FCRL5 delineates functionally impaired memory B cells associated with *Plasmodium falciparum* exposure. PLoS Pathog. 2015;11:e1004894. 25993340 10.1371/journal.ppat.1004894 PMC4438005

[90] Ehrhardt GRA . Expression of the immunoregulatory molecule FcRH4 defines a distinctive tissue‐based population of memory B cells. J Exp Med. 2005;202:783‐791. 16157685 10.1084/jem.20050879 PMC2212938

[91] Portugal S , Obeng‐Adjei N , Moir S , Crompton PD , Pierce SK . Atypical memory B cells in human chronic infectious diseases_ An interim report. Cell Immunol. 2017;321:18‐25. 28735813 10.1016/j.cellimm.2017.07.003 PMC5732066

[92] Obeng‐Adjei N , Portugal S , Holla P , et al. Malaria‐induced interferon‐γ drives the expansion of Tbethi atypical memory B cells. PLoS Pathog. 2017;13:e1006576‐e1006630. 28953967 10.1371/journal.ppat.1006576 PMC5633206

[93] Sundling C , Rönnberg C , Yman V , et al. B cell profiling in malaria reveals expansion and remodeling of CD11c+ B cell subsets. JCI Insight. 2019;4:126492. 10.1172/jci.insight.126492 PMC6538354 30939125

[94] Scholzen A , Teirlinck AC , Bijker EM , et al. BAFF and BAFF receptor levels correlate with B cell subset activation and redistribution in controlled human malaria infection. J Immunol. 2014;192:3719‐3729. 24646735 10.4049/jimmunol.1302960 PMC4028688

[95] Patgaonkar M , Herbert F , Powale K , et al. Vivax infection alters peripheral B‐cell profile and induces persistent serum IgM. Parasite Immunol. 2018;40:e12580‐e12614. 30102786 10.1111/pim.12580

[96] Nogaro SI , Hafalla JC , Walther B , et al. The Breadth, but not the magnitude, of circulating memory B cell responses to *Plasmodium* *falciparum* increases with age/exposure in an area of low transmission. PLoS ONE. 2011;6:e25582. 21991321 10.1371/journal.pone.0025582 PMC3186790

[97] Muellenbeck MF , Ueberheide B , Amulic B , et al. Atypical and classical memory B cells produce *Plasmodium falciparum* neutralizing antibodies. J Exp Med. 2013;210:389‐399. 23319701 10.1084/jem.20121970 PMC3570107

[98] Kim CC , Baccarella AM , Bayat A , Pepper M , Fontana MF . FCRL5+ memory B cells exhibit robust recall responses. Cell Rep. 2019;27: 1446‐1460e4. 31042472 10.1016/j.celrep.2019.04.019 PMC6530801

[99] Barnett BE , Staupe RP , Odorizzi PM , et al. Cutting edge: B cell‐intrinsic T‐bet expression is required to control chronic viral infection. J Immunol. 2016;197:1017‐1022. 27430722 10.4049/jimmunol.1500368 PMC4975981

[100] Rubtsova K , Rubtsov AV , van Dyk LF , Kappler JW , Marrack P . T‐box transcription factor T‐bet, a key player in a unique type of B‐cell activation essential for effective viral clearance. Proc Natl Acad Sci U. S. A. 2013;110:E3216‐E3224. 23922396 10.1073/pnas.1312348110 PMC3752276

[101] Winslow GM , Papillion AM , Kenderes KJ , Levack RC . CD11c+ T‐bet+ memory B cells: Immune maintenance during chronic infection and inflammation? Cell Immunol. 2017;321:8‐17. 28838763 10.1016/j.cellimm.2017.07.006 PMC5732029

[102] Racine R , Chatterjee M , Winslow GM . CD11c expression identifies a population of extrafollicular antigen‐specific splenic plasmablasts responsible for CD4 T‐independent antibody responses during intracellular bacterial infection. J Immunol. 2008;181:1375‐1385. 18606692 10.4049/jimmunol.181.2.1375 PMC2645789

[103] Jones DD , DeIulio GA , Winslow GM . Antigen‐driven induction of polyreactive IgM during intracellular bacterial infection. J Immunol. 2012;189:1440‐1447. 22730531 10.4049/jimmunol.1200878 PMC3401281

[104] Rubtsova K , Rubtsov AV , Halemano K , et al. T cell production of IFNy in response to TLR7/IL‐12 stimulates optimal B cell responses to viruses. PLoS ONE. 2016;11:1‐15. 10.1371/journal.pone.0166322 PMC5120817 27880772

[105] Wehr C , Eibel H , Masilamani M , et al. A new CD21low B cell population in the peripheral blood of patients with SLE. Clin Immunol. 2004;113:161‐171. 15451473 10.1016/j.clim.2004.05.010

[106] Frisullo G , Nociti V , Iorio R , et al. Increased expression of T‐bet in circulating B cells from a patient with multiple sclerosis and celiac disease. Hum Immunol. 2008;69:837‐839. 18940217 10.1016/j.humimm.2008.09.008

[107] Wang Z , Wang Z , Wang J , Diao Y , Qian X , Zhu N . T‐bet‐expressing B cells are positively associated with Crohn's disease activity and support Th1 inflammation. DNA Cell Biol. 2016;35:628‐635. 27348235 10.1089/dna.2016.3304

[108] Claes N , Fraussen J , Vanheusden M , et al. Age‐associated B cells with proinflammatory characteristics are expanded in a proportion of multiple sclerosis patients. J Immunol. 2016;197:4576‐4583. 27837111 10.4049/jimmunol.1502448

[109] Becker AM , Dao KH , Han BK , et al. SLE peripheral blood B cell, T cell and Myeloid cell transcriptomes display unique profiles and each subset contributes to the interferon signature. PLoS One.8:e67003. 23826184 10.1371/journal.pone.0067003 PMC3691135

[110] Rubtsov AV , Rubtsova K , Fischer A , et al. Toll‐like receptor 7 (TLR7)‐driven accumulation of a novel CD11c+ B‐cell population is important for the development of autoimmunity. Blood. 2011;118:1305‐1315. 21543762 10.1182/blood-2011-01-331462 PMC3152497

[111] Rubtsov AV , Rubtsova K , Kappler JW , Jacobelli J , Friedman RS , Marrack P . CD11c‐expressing B cells are located at the T cell/B cell border in spleen and are potent APCs. J Immunol. 2015;195:71‐79. 26034175 10.4049/jimmunol.1500055 PMC4475418

[112] Isnardi I , Ng Y‐S , Menard L , et al. Complement receptor 2/CD21‐ human naive B cells contain mostly autoreactive unresponsive clones. Blood. 2010;115:5026‐5036. 20231422 10.1182/blood-2009-09-243071 PMC3373152

[113] Rakhmanov M , Keller B , Gutenberger S , et al. Circulating CD21low B cells in common variable immunodeficiency resemble tissue homing, innate‐like B cells. Proc Natl Acad Sci U. S. A. 2009;1–6. 10.1073/pnas.0901984106 PMC2726348 19666505

[114] Rivera‐Correa J , Guthmiller JJ , Vijay R , et al. Plasmodium DNA‐mediated TLR9 activation of T‐bet+ B cells contributes to autoimmune anaemia during malaria. Nat Commun. 2017;8:1‐11. 29101363 10.1038/s41467-017-01476-6 PMC5670202

[115] Kenderes KJ , Levack RC , Papillion AM , Cabrera‐Martinez B , Dishaw LM , Winslow GM . T‐Bet+ IgM memory cells generate multi‐lineage effector B cells. Cell Rep. 2018;24:824‐837e3. 30044980 10.1016/j.celrep.2018.06.074 PMC6141031

[116] Defrance T , Taillardet M , Genestier L . T cell‐independent B cell memory. Curr Opin Immunol. 2011;23:330‐336. 21482090 10.1016/j.coi.2011.03.004

[117] Good‐Jacobson KL , Tarlinton DM . Multiple routes to B‐cell memory. Int Immunol. 2012;24:403‐408. 22451529 10.1093/intimm/dxs050

[118] Mewono L , Agnandji ST , Matondo Maya DW , et al. Malaria antigen‐mediated enhancement of interleukin‐21 responses of peripheral blood mononuclear cells in African adults. Exp Parasitol. 2009;122:37‐40. 19545527 10.1016/j.exppara.2009.01.007

[119] Obeng‐Adjei N , Portugal S , Tran T , et al. Circulating Th1‐cell‐type Tfh cells that exhibit impaired B cell help are preferentially activated during acute malaria in children. Cell Rep. 2015;13:425‐439. 26440897 10.1016/j.celrep.2015.09.004 PMC4607674

[120] Roetynck S , Olotu A , Simam J , et al. Phenotypic and functional profiling of CD4 T cell compartment in distinct populations of healthy adults with different antigenic exposure. PLoS ONE. 2013;8:e55195. 23383106 10.1371/journal.pone.0055195 PMC3557244

[121] Mewono L , Matondo Maya DW , Matsiegui P‐B , et al. Interleukin‐21 is associated with IgG1 and IgG3 antibodies to erythrocyte‐binding antigen‐175 peptide 4 of *Plasmodium falciparum* in Gabonese children with acute falciparum malaria. Eur Cytokine Netw. 2008;19:30‐36. 18299268 10.1684/ecn.2008.0114

[122] Pérez‐Mazliah D , Nguyen MP , Hosking C , et al. Follicular helper T cells are essential for the elimination of *Plasmodium* infection. EBioMedicine. 2017;24:216‐230. 28888925 10.1016/j.ebiom.2017.08.030 PMC5652023

[123] Wikenheiser DJ , Ghosh D , Kennedy B , Stumhofer JS . The costimulatory molecule ICOS regulates host Th1 and follicular Th cell differentiation in response to *Plasmodium chabaudi chabaudi* AS infection. J Immunol. 2016;196:778‐791. 26667167 10.4049/jimmunol.1403206 PMC4705592

[124] Zander R , Obeng‐Adjei N , Guthmiller J , et al. PD‐1 co‐inhibitory and OX40 co‐stimulatory crosstalk regulates helper T cell differentiation and anti‐plasmodium humoral immunity. Cell Host Microbe. 2015;17:628‐641. 25891357 10.1016/j.chom.2015.03.007 PMC4433434

[125] Laidlaw BJ , Lu Y , Amezquita RA , et al. Interleukin‐10 from CD4+ follicular regulatory T cells promotes the germinal center response. Sci Immunol. 2017;2:eaan4767. 29054998 10.1126/sciimmunol.aan4767 PMC5846620

[126] Guthmiller JJ , Graham AC , Zander RA , Pope RL , Butler NS . Cutting edge: IL‐10 is essential for the generation of germinal center B cell responses and anti‐ plasmodium humoral immunity. J Immunol. 2017;198:617‐622. 27940658 10.4049/jimmunol.1601762 PMC5225073

[127] Hahn WO , Butler NS , Lindner SE , et al. cGAS‐mediated control of blood‐stage malaria promotes *Plasmodium*‐specific germinal center responses. JCI insight. 2018;3:1‐18. 10.1172/jci.insight.94142 PMC5821207 29367469

[128] Crotty S , Kersh EN , Cannons J , Schwartzberg PL , Ahmed R . SAP is required for generating long‐term humoral immunity. Nature. 2003;421:282‐287. 12529646 10.1038/nature01318

[129] Qi H . From SAP‐less T cells to helpless B cells and back: dynamic T‐B cell interactions underlie germinal center development and function. Immunol Rev. 2012;247:24‐35. 22500829 10.1111/j.1600-065X.2012.01119.x

[130] Butler NS , Moebius J , Pewe LL , et al. Therapeutic blockade of PD‐L1 and LAG‐3 rapidly clears established blood‐stage *Plasmodium* infection. Nat Immunol. 2011;13:188‐195. 22157630 10.1038/ni.2180 PMC3262959

[131] Kurup SP , Obeng‐Adjei N , Anthony SM , et al. Regulatory T cells impede acute and long‐term immunity to blood‐stage malaria through CTLA‐4. Nat Med. 2017;23:1220‐1225. 28892065 10.1038/nm.4395 PMC5649372

[132] Szabo SJ , Kim ST , Costa GL , Zhang X , Fathman CG , Glimcher LH . A novel transcription factor, T‐bet, directs Th1 lineage commitment. Cell. 2000;100:655‐669. 10761931 10.1016/s0092-8674(00)80702-3

[133] Knox JJ , Kaplan DE , Betts MR . T‐bet‐expressing B cells during HIV and HCV infections. Cell Immunol. 2017;321:26‐34. 28739077 10.1016/j.cellimm.2017.04.012 PMC5732046

[134] Peng SL , Szabo SJ , Glimcher LH . T‐bet regulates IgG class switching and pathogenic autoantibody production. Proc Natl Acad Sci U. S. A. 2002;99:5545‐5550. 11960012 10.1073/pnas.082114899 PMC122806

[135] Coutelier JP , van der Logt JT , Heessen FW , Warnier G , Snick JV . IgG2a restriction of murine antibodies elicited by viral infections. J Exp Med. 1987;165:64‐69. 3794607 10.1084/jem.165.1.64 PMC2188250

[136] Markine‐Goriaynoff D , Coutelier J‐P . Increased efficacy of the immunoglobulin G2a Subclass in antibody‐mediated protection against lactate dehydrogenase‐elevating virus‐induced polioencephalomyelitis revealed with switch mutants. J Virol. 2002;76:432‐435. 11739710 10.1128/JVI.76.1.432-435.2002 PMC135718

[137] Dobaño C , Santano R , Vidal M , et al. Differential patterns of IgG subclass responses to *Plasmodium falciparum* antigens in relation to malaria protection and RTS. S Vaccination Front Immunol. 2019;10:13. 10.3389/fimmu.2019.00439 PMC6428712 30930896

[138] Roussilhon C , Oeuvray C , Müller‐Graf C , et al. Long‐term clinical protection from Falciparum Malaria is strongly associated with IgG3 antibodies to merozoite surface protein 3. PLoS Medicine. 2007;4:e320. 18001147 10.1371/journal.pmed.0040320 PMC2071934

[139] Zander RA , Vijay R , Pack AD , et al. Th1‐like *Plasmodium*‐specific memory CD4+ T cells support humoral immunity. Cell Rep. 2017;21:1839‐1852. 29141217 10.1016/j.celrep.2017.10.077 PMC5693336

[140] Ioannidis LJ , Nie CQ , Ly A , Ryg‐Cornejo V , Chiu CY , Hansen DS . Monocyte‐ and neutrophil‐derived CXCL10 impairs efficient control of blood‐stage malaria infection and promotes severe disease. J Immunol. 2016;196:1227‐1238. 26718341 10.4049/jimmunol.1501562

[141] Ryg‐Cornejo V , Ioannidis L , Ly A , et al. Severe malaria infections impair germinal center responses by inhibiting T follicular helper cell differentiation. Cell Rep. 2016;14:68‐81. 26725120 10.1016/j.celrep.2015.12.006

[142] Naradikian MS , Myles A , Beiting DP , et al. Cutting Edge: IL‐4, IL‐21, and IFN‐γ Interact To Govern T‐bet and CD11c Expression in TLR‐Activated B Cells. J. Immunol. 2016;197:1023‐1028. 27430719 10.4049/jimmunol.1600522 PMC4975960

[143] Yates JL , Racine R , McBride KM , Winslow GM . T cell‐dependent IgM memory B cells generated during bacterial infection are required for IgG responses to antigen challenge. J Immunol. 2013;191:1240‐1249. 23804710 10.4049/jimmunol.1300062 PMC3720767

[144] Manni M , Gupta S , Ricker E , et al. Regulation of age‐associated B cells by IRF5 in systemic autoimmunity. Nat Immunol. 2018;19:407‐419. 29483597 10.1038/s41590-018-0056-8 PMC6095139

[145] Zinöcker S , Schindler CE , Skinner J , et al. The V gene repertoires of classical and atypical memory B cells in malaria‐susceptible West African children. J Immunol. 2015;194:929‐939. 25556245 10.4049/jimmunol.1402168 PMC4297690

[146] Domeier PP , Chodisetti SB , Soni C , et al. IFN‐γ receptor and STAT1 signaling in B cells are central to spontaneous germinal center formation and autoimmunity. J Exp Med. 2016;213:715‐732. 27069112 10.1084/jem.20151722 PMC4854731

